# P2RX7 Purinoceptor as a Therapeutic Target—The Second Coming?

**DOI:** 10.3389/fchem.2018.00248

**Published:** 2018-06-28

**Authors:** Chris N. J. Young, Dariusz C. Górecki

**Affiliations:** ^1^Molecular Medicine Laboratory, Institute of Biomedical and Biomolecular Sciences, School of Pharmacy and Biomedical Sciences, University of Portsmouth, Portsmouth, United Kingdom; ^2^Faculty of Health and Life Sciences, The School of Allied Health Sciences, De Montfort University, Leicester, United Kingdom; ^3^The General Karol Kaczkowski Military Institute of Hygiene and Epidemiology, Warsaw, Poland

**Keywords:** ATP, cancer, DMD, inflammation, large pore, P2X7, P2RX7 KO mouse

## Abstract

The P2RX7 receptor is a unique member of a family of extracellular ATP (eATP)-gated ion channels expressed in immune cells, where its activation triggers the inflammatory cascade. Therefore, P2RX7 has been long investigated as a target in the treatment of infectious and inflammatory diseases. Subsequently, P2RX7 signaling has been documented in other physiological and pathological processes including pain, CNS and psychiatric disorders and cancer. As a result, a range of P2RX7 antagonists have been developed and trialed. Interestingly, the recent crystallization of mammalian and chicken receptors revealed that most widely-used antagonists may bind a unique allosteric site. The availability of crystal structures allows rational design of improved antagonists and modeling of binding sites of the known or presumed inhibitors. However, several unanswered questions limit the cogent development of P2RX7 therapies. Firstly, this receptor functions as an ion channel, but its chronic stimulation by high eATP causes opening of the non-selective large pore (LP), which can trigger cell death. Not only the molecular mechanism of LP opening is still not fully understood but its function(s) are also unclear. Furthermore, how can tumor cells take advantage of P2RX7 for growth and spread and yet survive overexpression of potentially cytotoxic LP in the eATP-rich environment? The recent discovery of the feedback loop, wherein the LP-evoked release of active MMP-2 triggers the receptor cleavage, provided one explanation. Another mechanism might be that of cancer cells expressing a structurally altered P2RX7 receptor, devoid of the LP function. Exploiting such mechanisms should lead to the development of new, less toxic anticancer treatments. Notably, targeted inhibition of P2RX7 is crucial as its global blockade reduces the immune and inflammatory responses, which have important anti-tumor effects in some types of malignancies. Therefore, another novel approach is the synthesis of tissue/cell specific P2RX7 antagonists. Progress has been aided by the development of *p2rx7* knockout mice and new conditional knock-in and knock-out models are being created. In this review, we seek to summarize the recent advances in our understanding of molecular mechanisms of receptor activation and inhibition, which cause its re-emergence as an important therapeutic target. We also highlight the key difficulties affecting this development.

## Introduction

P2RX7 is a 595aa protein belonging to the ionotropic purinergic P2X subfamily which consists of seven members, P2X1-7 (Burnstock and Knight, 2004). P2Xs represent an ancient form of purine chemical messenger reception, with relatives now cloned from species as diverse as *Homo sapiens* and *Dictyostelium* (Burnstock and Verkhratsky, 2012). All family members are trimeric ligand-gated ion channels displaying a preference for cations. Their subunits comprise intracellular N and C termini, two transmembrane domains and a large intervening extracellular region containing the ATP binding site (Surprenant et al., 1996). P2RX7, originally characterized by Cockcroft and Gomperts as the ATP^4−^ receptor in rat mast cells (Cockcroft and Gomperts, 1980) was previously also known by the name of P2Z receptor, responsible for the eATP-dependent lysis of macrophages (Surprenant et al., 1996). This confusion arose in part due to its many characteristics, which make this receptor entirely distinct from other P2Xs. These include uniquely lower affinity for eATP: EC_50_ >1 mM at physiological ion concentrations (Yan et al., 2010) and the ability to induce membrane blebbing and cell death. As such, P2RX7 is perhaps best known for its role in regulating innate and adaptive immune responses and is expressed on virtually all cell types of the immune system (Burnstock and Knight, 2017). Macrophages and microglia express high levels of P2RX7 (He et al., 2017; Young et al., 2017) and are perhaps the best studied cells in relation to receptor function both *in vitro* and *in/ex vivo* (Csóka et al., 2015). However, P2RX7 has a huge functional repertoire being involved in phenomena as diverse as inflammation (Rissiek et al., 2015), proliferation (Monif et al., 2010), migration and invasion (Qiu et al., 2014), metabolism (Amoroso et al., 2012), autophagy (Young et al., 2015), cell death (Massicot et al., 2013), and neurotransmission (Sperlágh et al., 2002). P2RX7 over-expression and over-activation have been implicated in numerous physiological/pathophysiological processes where, intriguingly, P2RX7 activation can result in both positive and negative outcomes depending on a host of factors such as intensity and duration of the agonist stimulus (Hanley et al., 2012), severity of pathogen virulence/infection (Figliuolo et al., 2017), the cell type (Cortés-Garcia et al., 2016; Young et al., 2017), extracellular ion concentration (Virginio et al., 1997), phospholipid membrane composition (Karasawa et al., 2017), co-factor activity (Migita et al., 2016), enzymatic processing (Young et al., 2017), polymorphic variations (Fuller et al., 2009; Ursu et al., 2014), and non-ATP agonist activation (Hong et al., 2009). The latter occurs during innate immune responses through the release of damage-associated molecular patterns (DAMPs; e.g., DNA, RNA, HMGB1, etc.) or pathogen-associated molecular patterns (PAMPs, e.g., LPS) either directly or via Toll-like receptors (TLRs). Specifically, TLR2 and TLR4 have been found to directly interact with P2RX7 via biglycan (Babelova et al., 2009). Classically, once eATP activates P2RX7, TLR4-mediated pro-IL-1β processing is followed by potassium efflux, NLRP3/ASC inflammasome assembly and caspase-1-dependent IL-1β maturation and release (Perregaux and Gabel, 1994; Pelegrin et al., 2008; Dubyak, 2012). Other P2RX7-dependent inflammatory activators include IL-6, ROS (Munoz et al., 2017), other caspases and MMPs (Gu and Wiley, 2006; Young et al., 2017). P2RX7 role in the adaptive immune response is to directly activate T cell populations, being a pre-requisite for IL-2 release, to orchestrate the balance between Treg and T helper cell populations, with receptor activation favoring the formation of T helpers and its blockade having the opposite effect (Schenk et al., 2011; Cekic and Linden, 2016). However, while its role in macrophages, microglia, and T cells has been researched extensively, our understanding of the role of P2RX7 in other immune cell types is still evolving. Neutrophils, for example, first thought devoid of P2RX7 expression in humans (Martel-Gallegos et al., 2010), have risen to the fore with the recent demonstration of neutrophil-mediated orchestration of the IL-1β/NLRP3 response in BMDNs (Karmakar et al., 2016). Exploration of tumor-associated immune cell populations has also led to the discovery that macrophages and microglia are not the only cells to express high levels of P2RX7. Tumors also display upregulated P2RX7 expression, reaching levels far exceeding that of macrophages (Young et al., 2017). In fact, all tumor types listed in The Cancer Genome Atlas were found to have upregulated levels of *P2RX7* expression, with sub-type-specific mutation patterns highlighting the significant potential for novel therapeutic approaches in this area (Young et al., 2017).

Compared to other P2X receptors, P2RX7 possesses an extra-long intracellular C-terminus with additional 200 amino acids. Mutations at various locations in this region have been associated with loss of diverse physiological functions such as LPS binding (Denlinger et al., 2001), post-translational modifications (Gonnord et al., 2009), membrane targeting (Smart et al., 2003) and large pore formation (Feng et al., 2006). P2RX7 stimulation at lower-end eATP doses opens a cation-selective plasma membrane channel permeable to Ca^2+^, Na^+^, and K^+^. Chronic/sustained eATP dosing reveals the second facet to P2RX7 signaling; the formation of a non-selective large pore (LP), permeable to molecules of up to 900 Da, such as dyes (EtBr, 314 Da; YO-PRO-1, 376 Da and Lucifer Yellow, 457 Da Adinolfi et al., 2005). Thus, P2RX7 can display the properties of a prototypic cytotoxic receptor. The inherent insensitivity to eATP coupled with dire consequences of receptor over-stimulation prompted the belief that this low agonist affinity might be a safe-guard against potentially damaging receptor-mediated inflammatory cascade activation through the release of IL-1β, additional eATP or other DAMPs. Indeed, although healthy interstitial eATP levels are maintained at nanomolar levels or below, it has been shown that the eATP concentration around sites of inflammation can increase several fold (Morciano et al., 2017). For example, eATP around damaged cells or dystrophic muscle fibers can reach 5–10 mM (Di Virgilio, 2000; Hetherington et al., 2001) and in the tumor microenvironment, levels of >700 μM have been recorded (Pellegatti et al., 2008). The tonic, low-level vs. chronic high-level receptor stimulation have very different outcomes, indeed. Moreover, the balance and fine tuning of the molecular controls that govern these responses may underlie some of the most exciting new possibilities for therapeutic manipulation. Efforts in this area have been hampered by the lack of a P2RX7 crystal structure. However, recent successful crystallizations and 3-D reconstructions include both the 356 aa (without the C-terminal tail) Panda (*Ailuropoda melanoleuca)* pdP2RX7 (Karasawa and Kawate, 2016) as well as the truncated variant (lacking 27 N- and 214 C-terminal aa residues) of the chicken ckP2RX7 (Kasuya et al., 2017). These developments permit rational mechanistic studies (Karasawa and Kawate, 2016) with exciting novel insights into the specific mechanisms of both permeability and drug interactions. Appreciating the number of recent reviews on P2RX7 as a therapeutic target (Burnstock and Knight, 2017; Di Virgilio et al., 2018a; Savio et al., 2018) in this paper we discuss recent developments in P2RX7 drug design and repurposing, highlighting the potential new therapeutic applications of these drugs in human disorders.

## Recent developments in P2RX7 crystallography

**Figure 1:**
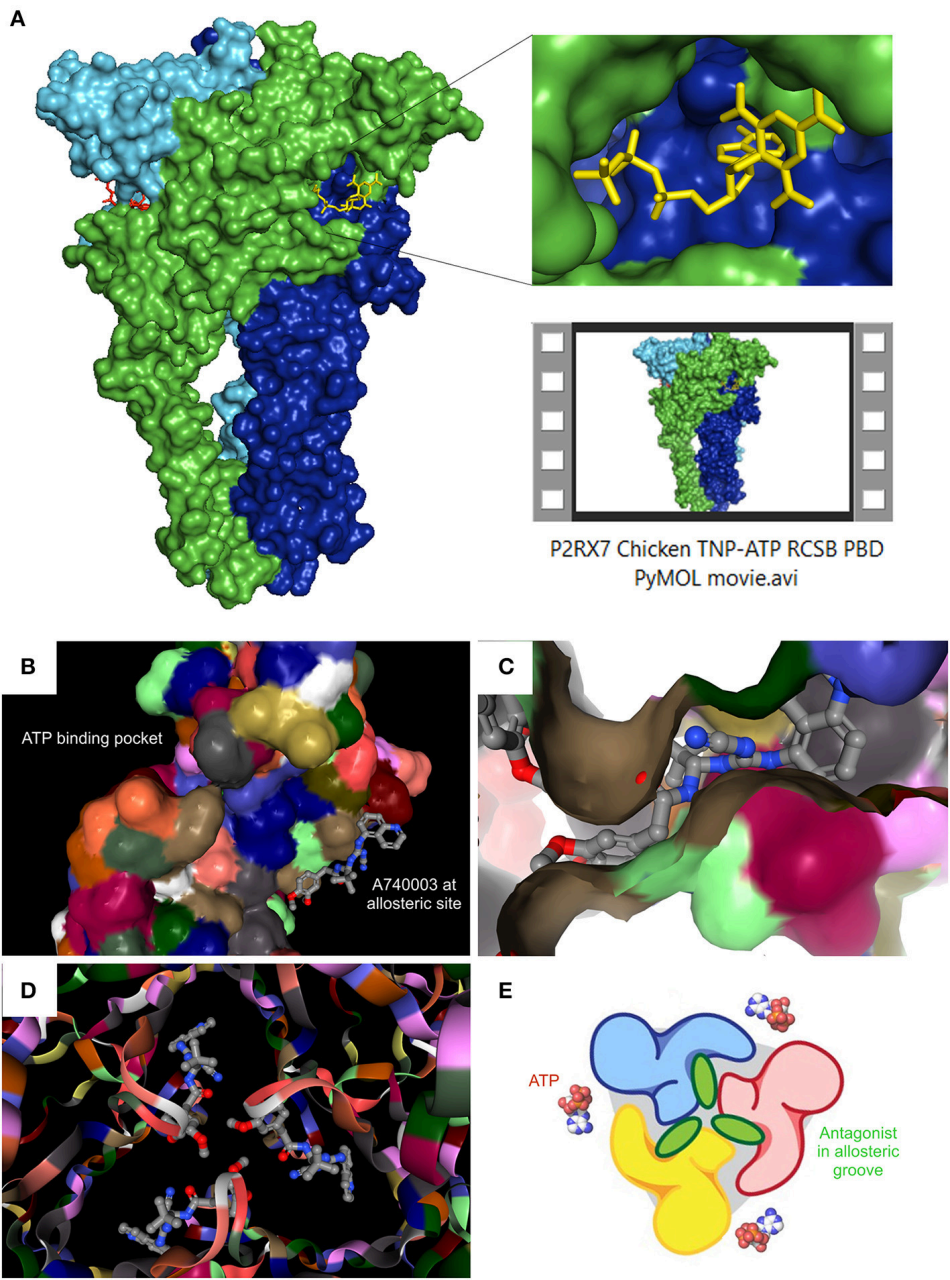
Recent P2RX7 structural developments: the ATP binding pocket and the allosteric groove. **(A)** PyMOL (pymol.org) generated surface plot of P2RX7 trimer using RCSB PDB data file (rcsb.org) for chicken variant bound to the competitive antagonist TNP-ATP (structure 5XW6, 3.1A). ROI shows the ATP binding pocket (RHS, upper), PyMOL Video file gives structural overview of the trimer with detailed orientations of the ATP binding pocket and the central ion channel (see Supplementary Material Video S1). RCSB PDB generated images show location of the ATP binding pocket in relation to the newly discovered allosteric site on the monomer **(B)** and at the subunit interface of the trimer, forming an allosteric groove—here shown occupied by A740003 antagonist **(C)**. When imaged together from intracellular projection, the three allosteric grooves appear as a Shuriken, whose rotation is impeded by the allosteric antagonist A740003 **(D)**. **(E)** Cartoon representation of the relationship between the agonist binding site and the allosteric antagonist-binding groove. Adapted from Karasawa and Kawate (2016), with permission.

Crystal structures for zebrafish P2RX4 (Kawate et al., 2009), then human P2RX3 (Mansoor et al., 2016) gave the first insights into conserved mechanisms of action: the ATP binding pocket, subunit interactions and conformation changes upon agonist binding (i.e., predictions of gating and opening) (Hattori and Gouaux, 2012). Individual subunits of these crystallized receptors (zebrafish and human alike) adopt “dolphin-shaped” conformations (see Kawate et al., 2009; Karasawa and Kawate, 2016). The aforementioned recent crystallizations of both ckP2RX7 (Kasuya et al., 2017) and pdP2RX7 (Karasawa and Kawate, 2016) confirmed this structure. 3-D reconstructions of trimeric subunit interactions also confirm three equivalent ATP binding site locations at subunit interfaces. More excitingly, the pdP2RX7 structure has revealed a completely novel allosteric site situated in the groove formed between two adjacent subunits, which is completely distinct from the ATP binding site (Karasawa and Kawate, 2016). ckP2RX7 and pdP2RX7 share ~62 and 85% homology with human P2RX7A (Karasawa and Kawate, 2016), respectively. Unfortunately, both crystal structures lack the C-terminal, intracellular tail region, so important for the receptor function (see below). Achieving the complete P2RX7 3-D structural resolution remains an unclaimed trophy. However, even the partial pdP2RX7 structure has revealed some unexpected features. When expressed in liposomes, the pdP2RX7 triggered LP formation despite lacking the C-terminus. The proposed mechanism was the regulatory dependence of the P2RX7 large pore on the membrane lipid composition, where the cholesterol level played a significant role: increases in membrane cholesterol correlated with reductions in membrane fluidity and LP formation in this liposome-based system (Karasawa et al., 2017). The singularly unique aspect of the pdP2RX7 3-D modeling however, was the discovery of a new allosteric binding site, distinct from the ATP-binding pocket, at each subunit interface. The structurally unrelated antagonists A740003, A804598, AZ10606120, GW791343, and JNJ47965567 all were shown to bind this allosteric “groove” between subunit faces (Karasawa and Kawate, 2016). Kawate et al., used functional fluorescence anisotropy-based assays to show that these compounds effectively act as “door-stops” or “wedges,” preventing the narrowing of this groove during the turret-like conformational rotation during channel dilation upon agonist binding. Mutational replacement of residues with “bulkier” cysteines resulted in effective narrowing of the inter-subunit groove and reduced channel opening kinetics. Thus, we now know that it is the spatial filling of this new inter-subunit non-competitive hydrophobic pocket, which is the determining factor in antagonist selectivity and potency. The pocket itself is composed of β-strand residues (β4, β13, and β14) with hydrophobic interactions at positions deep inside the pocket (F95, F103, M105, F293, and V312) (Karasawa and Kawate, 2016). See Figure 1 for structural representations of the eATP binding pocket and the allosteric antagonist binding groove.

The remaining limitation is the structural resolution (~3.2~3.6 Å), which does not currently facilitate definitive predictions of bond angles or side chain-drug interactions. However, the main dimensions of the pocket are clearly defined and this finding have opened the door for significant strategic antagonist developments through targeted screening and rational design approaches. Moreover, with the apparent absence of this allosteric site in the known P2RX3 and P2RX4 crystal structures, there is a potential for the development of highly P2RX7-selective drugs. However, since ATP binding was also found to narrow the groove (Karasawa and Kawate, 2016), further functional studies are required to determine the accessibility of this allosteric site under conditions of high eATP concentration. Hence, the groove might theoretically be less accessible due to continued receptor activation, which could have implications for therapeutic applications. Moreover, positive allosteric agonists such as polymyxin B (Ferrari et al., 2004) may share this novel allosteric site or indeed may occupy other distinct sites. Such intricacies remain unresolved but the crystal structure makes further studies possible.

## P2RX7 kinetics: channel becomes pore?

It was the 3-D structure predictions of P2RX4, which first suggested ion flux upon agonist binding to be initiated through the rotation of six helical transmembrane domains of the trimeric receptor (Hattori and Gouaux, 2012). Once TM2 Val335-Leu346 moves, the channel gate is opened and current may flow as directed by the membrane potential (Li et al., 2008). However, P2RX7 ion flux studies at the upper-end eATP concentrations (typical for damaged tissues) are hampered by what appears to be the biphasic nature of the channel, leading to a second mode of opening. Given that pathological P2RX7 responses have been linked to the activation of this large pore, this has been a singularly enigmatic and controversial issue in the P2RX7 field. Chronic receptor activation under the appropriate conditions unquestionably facilitates massive membrane depolarization, dye permeability and, in some circumstances, cell death via mechanisms that might be cell-specific and even unique (e.g., pyroptosis, autosis etc.). However, the pore opening is reversible. Moreover, mechanism(s) behind these effects remained elusive. Two very mechanistically distinct hypotheses tried to explain the large pore phenomenon. One that P2RX7, under conditions of chronic eATP stimulation, recruits a secondary pore complex. The fact that P2RX7 expression alone in Xenopus oocytes proved insufficient to induce pore formation in these cells seemingly supported the role of either some accessory protein or other as yet unknown accessory mechanism (Petrou et al., 1997), with pannexin1 (Panx1) channels being pointed out as the main candidate (Pelegrin and Surprenant, 2006). The second hypothesis is that the P2RX7 trimer can further change conformation resulting in ion channel to pore dilation (Browne et al., 2013). The LP-through-Panx1 theory is complex in its regulatory constitution, with multiple splice variants and cell-specific expression patterns to contend with (Ma et al., 2009). Moreover, there is evidence that the P2RX7 LP formation is retained in Panx1^−/−^ cells (Qu et al., 2011; Hanley et al., 2012; Alberto et al., 2013) and that the P2RX7 trimer can form this LP structure alone (Browne et al., 2013; Karasawa et al., 2017). However, the existence of P2RX7 in two opening states was not supported by recent single-channel electrophysiological studies, which provided no evidence for dilation of the hP2X7R channel on sustained eATP stimulation (Pippel et al., 2017). Indeed, it was the discrepancy between the receptor activation data obtained by whole-cell vs. single-channel currents and via dye permeability, which triggered the notion that the LP must involve an additional molecular entity. Interestingly, a recent review seems to reconcile some of the seemingly contradictory findings (for a comprehensive discussion see Di Virgilio et al., 2018b). The authors argue that the model of channel-to-pore transition might have resulted from the different kinetics of fluorescence increases evoked by Ca^2+^ sensors in response to calcium influx (rapid) and the florescence from DNA dye binding (slow). Consequently, the LP formation may occur concomitantly with the opening of the ion channel and the two signals increasing over distinct time-scales. This model fits with recent data that the P2RX7 pore can dilate sufficiently to allow Yo-Pro permeation (Karasawa et al., 2017). However, as specific mutations (the nfP2RX7) or splicing affecting the C-terminus of P2RX7 abolish LP dye uptake but not ion channel functions, we may not have the full explanation yet. The picture should become clearer as we move closer to a complete 3-D crystal structure determination and understand the receptor interactome better. For instance, demonstration of cholesterol/membrane lipid-specific controls over pore dilation (Karasawa et al., 2017) could explain the inherent difficulties associated with successful patch-clamping involving the LP. The physical act of clamping a locality of the cell membrane may simply introduce sufficient rigidity to inhibit phospholipid movement in the vicinity of P2RX7. No doubt, this debate will continue, resulting in some ingenious technical development before the final conclusion is reached.

**Figure 2:**
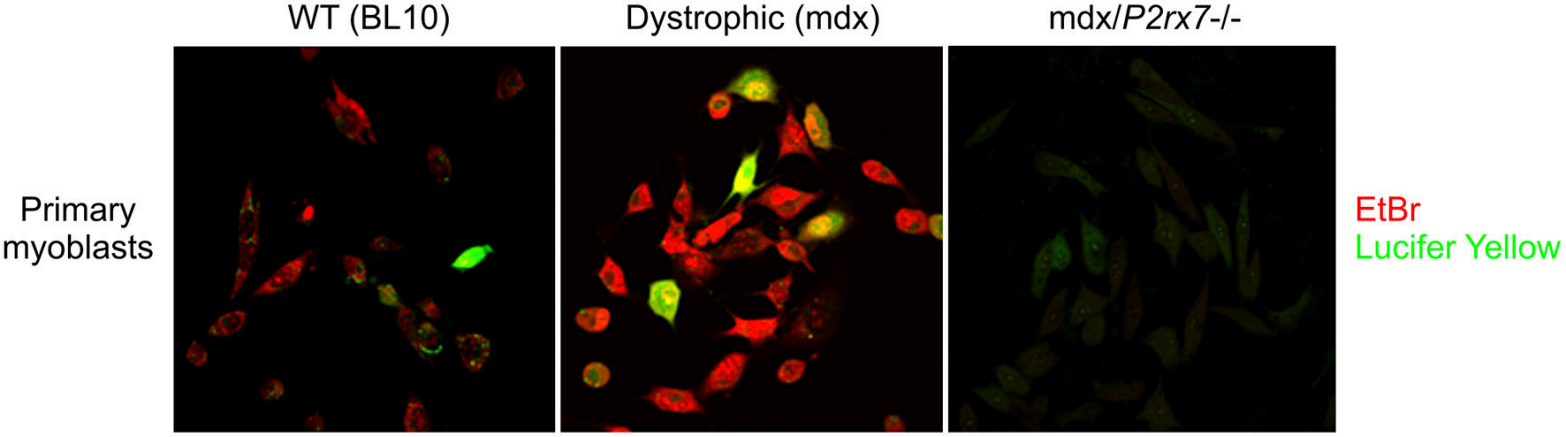
Selective regulation of cationic and anionic dye permeation through the P2RX7 LP. Example of differences in P2RX7 LP permeation preferences to EtBr (cationic) and Lucifer Yellow (anionic) dyes in individual cells of a clonal population (Young and Gorecki, 2016): Compared to WT myoblasts **(Left)**, the dystrophic mdx myoblasts **(Center)** display upregulated P2RX7 expression and increased LP permeability in response to eATP (Young et al., 2015, 2017). The mdx/P2RX7^−/−^ myoblasts **(Right)** show no dye uptake under these conditions, as expected. Note that the majority of the seemingly identical cells in this clonal population shows a clear preferential uptake of EtBr over LY upon the LP opening, which is considered to be a non-selective pore. The molecular mechanisms underlying this phenomenon are currently under investigation as this experiment illustrates the potential for diverse LP responses in heterogeneous and functionally plastic populations of e.g., immune cells *in vivo*.

An additional level of complexity exists around the selectivity of the P2RX7 LP, which displays a preference for cations or anions under different physiological conditions and in different cell types as well as in individual cells within a clonal population: In P2RX7 transfected HEK293 cells, Lucifer Yellow (LY) uptake displays intracellular Ca^2+^ dependence at rat P2RX7, but not mouse P2RX7 expressed in Raw264.7 cells. In the same study, EtBr uptake showed no such dependence (Cankurtaran-Sayar et al., 2009). In macrophages, no such calcium dependence occurs, but distinct pathways do exist, as evidenced by preferential uptake of cationic or anionic dyes by individual cells < 100 microns apart in the same dish (Schachter et al., 2008; Cankurtaran-Sayar et al., 2009). Our own studies have confirmed this uptake selectivity in dystrophic myoblast populations (Figure 2). Analysis of the fluorescent signal kinetics of LY vs. DNA binding dyes might be useful as the latter binds irreversibly and the difference in the dye uptake might be a reflection of cell health rather than the P2RX7 permeability function. Undoubtedly, understanding this mechanism might lead to tailored therapeutic approaches, with a particular scope in tumor cells.

Furthermore, irrespective of the mechanistic unknowns and controversies, the real ambiguity had always been the physiological relevance and function of this P2RX7 LP. The first demonstration of a functional link has been identification of a reciprocity between P2RX7 LP opening and autophagosome formation, where the one is indispensable for the other (Young et al., 2015). Given the recent progress in our understanding of the importance of autophagy-dependent processes in physiology and a range of human pathologies as diverse as neurological /neurodegenerative diseases (Alzheimer's, dementia, Parkinson's, and MLS), muscular dystrophies, infectious, lysosomal storage diseases, Crohn's, cancers and aging (Young et al., 2009, 2015; Rubinsztein et al., 2011; Nikoletopoulou et al., 2015; Qian et al., 2017), this link may suggest a potential for tuning autophagic responses in diverse human pathologies using P2RX7 modulation.

The relationship between pore formation and cell death is complex and also remains unclear; is pore formation a pre-requisite for P2RX7-dependent cell death? Given the controversies over the question whether the influx of ions through the channel is observed prior to the pore formation, we do not have a full answer. Working with these two facets involves independent conditions and very different experimental setups. The channel is mostly measured by electrophys and the LP by dye uptake. The sensitivity difference inherent to these methods is significant and generally, channel activity is recorded within seconds, whilst LP opening follows up to an hour later. Since no pharmacological antagonists truly specific to the LP has been identified, it is currently impossible to block the pore formation without blocking the channel. Hence, P2RX7-dependent death might be attributed to the channel without verifying pore functions (Kong et al., 2005). Whilst P2RX7-dependent, pore-independent cell death has been suggested in embryonic neural progenitor cells (Delarasse et al., 2009), this experiment was carried out using unusually high (2.5 mM) BzATP concentration. In contrast, P2RX7 activation with 100 μM BzATP was shown to induce differentiation in embryonic neural progenitor cells in N2-containing media, where pore formation and cell death were again not observed (Tsao et al., 2013). Yet, 300 μM has been shown to induce pore formation and cell death in adult neural progenitors in artificial cerebral spinal fluid (Messemer et al., 2013). This example illustrates difficulties in drawing conclusions around LP formation being a prerequisite for cell death as slightly different parameters will have a significant effect on experimental outcomes. Our own data suggest that serum content has a significant impact on the pore function (Young et al., 2017). This factor does not affect channel functions and is not always described in assay methodologies. The difference in P2RX7 receptor density on various cells and in the sensitivity of respective detection methods may also skew the analyses of channel and pore responses. Moreover, it is commonplace for one P2RX7 activation mode to be studied without the other. Hence, it is often difficult to make cross-study comparisons.

## P2RX7 localisation, signaling, interactome, and regulation

P2RX7 is a membrane-spanning receptor. Targeted mutagenesis has implicated the C-terminal tail region in this receptor trafficking to the membrane (reviewed in Robinson and Murrell-Lagnado, 2013). Interestingly, nuclear P2RX7 localisation has also been reported (Atkinson et al., 2002), with suggested roles in modulating neuronal plasticity, albeit no follow-up studies supported this finding. The association of P2RX7 with lipid rafts has been demonstrated in various systems (Garcia-Marcos et al., 2006) and may be associated with palmitoylation of C-terminal cysteine residues (Gonnord et al., 2009), which is a reversible modification associated with membrane targeting. This is intriguing given the more recent demonstration of cholesterol-mediated inhibition of both P2RX7 LP (Karasawa et al., 2017) and channel (Murrell-Lagnado, 2017), cholesterol being a significant constituent of lipid rafts. The idea that P2RX7 is both targeted to and could be inhibited by lipid raft cholesterol, suggests an as yet un-characterized mechanism of spatial regulation through lipid raft targeting. Interestingly, multiple tumor types show upregulation of lipid rafts and cholesterol-depleting agents were shown to induce apoptosis in such cells (Li et al., 2006). The existence of a P2RX7-evoked cell death cascade silencing through cholesterol regulation in cancer is an interesting possibility.

In this context of cancer cell targeting, the ‘non-functional' P2RX7 (nfP2RX7) (Slater et al., 2004) is the subject of numerous patents for epitope-specific antibody-based therapy for several cancer types (Gilbert et al., 2017). The nfP2RX7, in fact, retains the calcium channel functions, which are used by cancer cells to support its growth but lacks the ability to form the large pore and therefore not able to trigger cell death in the high eATP environment. This conformational change might be a specific mechanism controlling the potentially deleterious LP opening and allowing cancer cells expressing high P2RX7 levels to survive in the high eATP environment.

Furthermore, a feedback loop has been discovered, where sustained activation of P2RX7 with LP opening causes release of active MMP-2, which halts responses *via* the MMP-2-dependent receptor cleavage (Young et al., 2017). This mechanism operates in diverse cells, including macrophages, dystrophic myoblasts, P2RX7-transfected HEK293 and also in cancer cells. This tuning mechanism allows malignant cells to profit from P2RX7 channel activation eliciting proliferation, growth, migration, invasion and metabolic advantages, whilst avoiding cell death cascades associated with the LP. Moreover, MMP-2 is found in serum, where it displays complex regulation via TIMP expression/activity. Therefore, P2RX7 in organs with discontinuous capillaries, or in pathologies affecting capillary permeability (e.g., inflammation or tumor neo-vascularisation) may be under the regulatory control of MMP-2 cleavage balanced by TIMPs. CD44 has been reported to allosterically positively modulate P2RX7 activity by facilitating eATP binding through GAG chain interactions (Moura et al., 2015). CD44 itself was found to be a proteolytic target of MMP-2 (Young et al., 2017), suggesting that an additional levels of regulatory control may also exist. Given that MMP-2 inhibition can re-open the P2RX7 LP in cancer cells and effectively switch on the LP-associated cell death pathway, it might be possible to develop a new generation of cancer therapeutics promoting this P2RX7 LP formation (Young et al., 2017). Furthermore, as P2RX7 ablation eliminated gelatinase activity *in vivo*, P2RX7 antagonists could be a good alternative to highly toxic MMP inhibitors in treatments of inflammation and cancers.

Interestingly, non-eATP P2RX7 agonists also exist. NAD^+^ is perhaps the best studied here: ADP-ribosyltransferase (ART) enzymes catalyze the transfer of ADP-ribose groups from NAD^+^ to arginine residue 125 of the ecto-domain lying proximal to the P2RX7 ATP binding site (Seman et al., 2003). Ribosylations are covalent modifications and therefore constitutively activate P2RX7. However, this mode of P2RX7 activation appears to be species-specific, acting on mouse P2RX7 receptor variants, but not human. One rationale is that the Art2b gene encoding ART2.2 ADP-ribosyl transferase is differentially expressed between human and mouse; mice have two active copies of this gene, whereas in humans the transcript is translationally silenced by three premature stop codons (Haag et al., 1994). Moreover, this mechanism of activation is known to function in mouse T lymphocytes but not macrophages, the latter only display augmentation of eATP-induced receptor activation in the presence of NAD^+^ (Hong et al., 2009). Therefore, this effect may also be cell or isoform specific. Mouse but not human has two N-terminal isoforms and the unique P2RX7k variant is prominent in mouse T lymphocytes and particularly sensitive to ADP-ribosylation (Rissiek et al., 2015). Therefore, it might be the mouse-specific P2RX7k isoform responding to NAD^+^ stimulus. Indeed, in the Glaxo knockout, which retains the P2RX7k variant, receptor responses in T lymphocytes are retained (see P2X7 knockout section below). Finally, ADP-ribosyltransferase enzymes also display tissue specific expression patterns, which would also contribute to the regulation of NAD^+^ P2RX7 signaling.

While other non-nucleotide agonists of P2RX7 have been reported, the mode of their receptor interaction has not been determined and may either represent competitive or allosteric agonist/modulation. The recent structural analyses seem to favor the latter, given the steric constraints of the ATP-binding pocket. The immunopeptides amyloid β (Sanz et al., 2009) and serum amyloid A (Elssner et al., 2004), along with LL-37 (Elssner et al., 2004), and polymyxin B (Ferrari et al., 2004) have all been implicated in activating/potentiating the P2RX7 channel function through direct interactions with the receptor extracellular loop region. Cytoplasmic agonists have also been postulated, although these are indirect in nature: LPS binding P2RX7 C-terminal tail was shown to lower the stimulus intensity required for channel gating through eATP release (Yang et al., 2015). Cytoplasmic levels of Alu-RNA were suggested to function in the same manner, yet without inducing eATP release (Fowler et al., 2014).

Numerous cell-specific downstream signaling pathways are linked with P2RX7 activation. Those associated with the release of inflammatory mediators: caspase-1, IL-1β, IL-6, and NLRP3/ASC activation/recruitment have already been described. P2RX7 is also a key modulator of various signaling cascades involved in many physiological processes, including PKC-MEK-ERK-FOS-JUN (Tsao et al., 2013), PI3K-AKT-mTOR (Bian et al., 2013), MyD88-NFKB (Liu et al., 2011), MMP-2/9-Dystroglycan-CD44 (Young et al., 2017), and Calcineurin-NFATc1 (Shiratori et al., 2010). These numerous cascades illustrate the diversity and complexity of P2RX7 signaling, with implications for cellular processes such as growth, proliferation, differentiation, metabolism, migration, invasion, autophagy and also cell death. Mechanisms of P2RX7-dependent cell death have been well documented and are numerous, including apoptosis (Zanovello et al., 1990) as well as several unique processes: aponecrosis (MacKenzie et al., 2001), necroptosis (Cullen et al., 2015), pyroptosis (de Gassart and Martinon, 2015), pseudo-apoptosis (Roger and Pelegrin, 2011), and autosis (Draganov et al., 2015). Autophagic cell death has also been identified (Young et al., 2015).

The P2RX7 interactome is just as diverse. This receptor has been suggested to directly interact with over 50 different binding partners, most of which have been identified by combinations of mass spectrometry, immunoprecipitation and immunoblotting. P2RX7 interactors include soluble, membrane-bound, peripheral, cytoskeletal and chaperone proteins (summarized in P2X7.co.uk). This diversity of proteins may reflect further spatial and temporal complexity. Soluble biglycan for example, a ubiquitous extracellular matrix (ECM) proteoglycan is responsible for coordinating the interaction of P2RX7, TLR4, and P2RX4, facilitating NLRP3 inflammasome formation and IL-1β release (Babelova et al., 2009). When proteolytically activated in the ECM, biglycan itself becomes a danger signal, with the capacity to orchestrate both sterile and infectious inflammation (Nastase et al., 2012). Interestingly, biglycan has been studied extensively in muscle disorders, where P2RX7 upregulation and role in both inflammatory and non-inflammatory processes has also been established (Young et al., 2012, 2013, 2015; Sinadinos et al., 2015). Thus, tissue- and cell-specific modalities, as well as expression, localization and activation of binding partners plus influence of inflammatory mediators, all play a role in P2RX7 signaling.

## P2RX7 agonists and antagonists: where are we?

As already explained, eATP is able to induce different gating characteristics depending on concentrations around the active site. Essentially, eATP >100 μM is sufficient to activate the channel and >100–300 μM and above are necessary to induce LP formation (Donnelly-Roberts et al., 2009). Endogenous eATP is subject to degradation by ubiquitous extracellular nucleotidases such as CD73 and CD39, as well as tissue-specific enzymes, such as α-sarcoglycan (adhalin) found in skeletal muscles (Betto et al., 1999). In this way, eATP concentrations in the extracellular space are tightly regulated, where high concentrations are quickly degraded to ADP, AMP and adenosine, which activate other P2X, P2Y, and P1 receptors, respectively. Importantly, activation of these different P1 and P2 receptors can have opposite effects to eATP stimulation, thus creating yet another level of homeostasis but is also confounding pharmacological studies. Moreover, conditions where surface nucleotidases become deregulated, such as in the case of loss of adhalin from the muscle sarcolemma in Duchenne and limb-girdle muscular dystrophies, result in elevated levels of eATP on the cell surface (Betto et al., 1999). In such conditions, changes in the receptor activity might be unrelated to its properties or expression levels. Therefore, P2RX7 specific and non-hydrolysable agonists would be useful tools for dissecting distinct P2RX7-specific downstream responses. While the competitive agonist benzoylated-ATP (BzATP) and non-hydrolysable sulfonated-ATP (ATPγS) display increased specificity and affinity over endogenous eATP, no true P2RX7-specific agonist has been identified so far. Hence, BzATP, with 10-30 times higher affinity for channel gating at the human P2RX7 receptor is the most widely used (Bianchi et al., 1999) despite its known activity at P2RX1 and P2RX3 and being metabolized to lower MW constituents (De Marchi et al., 2016). Species-specific differences in agonist specificity and sensitivity add an additional level of complexity to pharmacological studies (Hibell et al., 2000).

P2RX7 blockers are currently divided into two main mechanistic groups: one which binds orthosterically and competitively to the eATP binding pocket and another that binds allosterically to sites other than the eATP site and reduces ligand binding affinity. First generation P2RX7 antagonists were developed in the 1990s and can now be grouped according to their lack of specificity. Examples include Reactive Blue 2, Suramin (and its derivatives), Coomassie Brilliant Blue G (BBG), pyridoxal phosphate-6-azophenyl-2-4-disulfonic acid (PPADS), 1-N,O-bis(5-isoquinolinesulfonyl)-N-methyl-l-tyrosyl-4-phenylpiperazine (KN-62), and oxidized ATP (oATP), see Bartlett et al. for a review (Bartlett et al., 2014). Despite its “non-druggable” nature and lack of absolute specificity for P2RX7 and capacity for near-infra red fluorescence upon protein binding, BBG remains one of the most widely used and useful reagents as it is effective at human, mouse, rat, dog and guinea pig P2RX7. BBG also had triggered the initial interest in therapeutic applications by showing effectiveness in preventing eATP-induced cell death in murine models of spinal cord injury *in vivo* (Wang et al., 2004; Peng et al., 2009) and because of its blood-brain barrier (BBB) permeability. Indeed, despite its limitations, BBG has been used widely to block P2RX7 in various pathologies such as neuroexcitotoxicity (Carmo et al., 2014), graft-vs.-host disease (Geraghty et al., 2017) and muscular dystrophy (Young et al., 2012). However, as BBG is not a specific P2RX7 antagonist, also inhibiting P2RX4 and P2RX1 receptors, the pharmacological findings obtained with this antagonist have been confirmed using *p2rx7* KO models. Two of these are available and have been used in the bulk of studies: The GSK knockout carries a lacZ/Neo cassette insertion in exon 1 (Sikora et al., 1999; Chessell et al., 2005) and Pfizer's (available via JAX) has a Neo cassette insertion in exon 13 (Solle et al., 2001). Importantly, in both models, *P2rx7* gene escaped complete inactivation, retaining expression of some isoforms P2RX7k in Glaxo (Nicke et al., 2009) and P2RX7a and P2RX7b in Pfizer (Masin et al., 2012). Specifically, T cells and lymphocytes from the Glaxo KO have been shown to retain P2RX7 responses, including LP function (Taylor et al., 2009). As discussed above, this is due to the existence, in the mouse, of the P2RX7k variant, which is driven by an alternative promoter and its exon 1 escaping inactivation in the GSK model. This isoform, although expressed at low levels, displays faster activation kinetics and a lower threshold for channel gating in comparison to the wild-type P2RX7a variant (Nicke et al., 2009). The Pfizer KO mouse retains expression of the alternative C-terminal variants, however these have limited homomeric functionality and low expression levels in most if not all cell types. Hence, while the Pfizer model can be regarded as a functional knockout, studies using the GSK animal should be carefully re-considered and interpreted appropriately. Recently, new mouse models have been developed, including some allowing the conditional, cell-type specific *p2rx7* knockout. These mice should be an invaluable resource for deciphering the cell, tissue-specific and temporal effects of P2RX7 receptor activation and inhibition.

Other groups of functionally related molecules have also been shown to block P2RX7: Naturally-occurring plant compounds such as alkaloids (Shemon et al., 2004), synthetic enzyme inhibitors such as CAY10593 (phospholipase D blocker) (Pupovac et al., 2013), antipsychotics (Hempel et al., 2013) as well as various cations, such as Ca^2+^ and Mg^2+^ (Jiang, 2009). While the first-generation compounds such as oATP and BBG are still being used in some experimental paradigms, second-generation P2RX7 antagonists offer significantly increased specificity, albeit at significantly increased cost. This second generation antagonists began to emerge in 2006 with the development by Abbott Labs of disubstituted tetrazoles such as A438079 (Nelson et al., 2006) and cyanoguanidines such as A740003 (Honore et al., 2006). Interestingly, both classes were previously thought to act reversibly and competitively (Donnelly-Roberts and Jarvis, 2007). However, recent structural data contradicted this assumption by placing A74003 not in the ATP-binding pocket but acting via the newly identified allosteric, non-competitive site instead (Karasawa and Kawate, 2016). Intriguingly, A438079 binding analysis is not yet available while this compound appears to preferentially block the LP (Haanes et al., 2012). It would be therefore interesting to interrogate the newly available crystal structure to determine whether A438079 and A740003 share the same binding site as this information may help our understanding of the channel vs. pore functionality. Another important step has been the development of a stable, selective orally bioavailable, blood brain barrier permeable P2RX7 antagonists suitable for the treatment of a host of human pathologies. High-throughput screening has led to the identfication of Glaxo GSK314181A (Broom et al., 2008) and AstraZeneca AZ11645373 (Stokes et al., 2006, also see Mehta et al., 2014 and Syed and Kennedy, 2012 for extensive reviews (Syed and Kennedy, 2012; Mehta et al., 2014) followed by AZD9056, also from AstraZeneca (Keystone et al., 2012) and CE-224,535 from Pfizer (Stock et al., 2012). Since the development of these new drugs, pharmacological inhibition of P2RX7 has proven to be effective and well-tolerated treatment in multiple rodent models of inflammatory diseases including neurological, musculoskeletal as well as retinal, kidney, bladder, liver, lung, and skin-based inflammatory disorders (reviewed extensively in Bartlett et al., 2014, in models of stroke, brain trauma, hemorrhage and also in BBB integrity loss Zhao et al., 2016) as well as muscular dystrophy, cardiomyopathy, respiratory disorders, inflammatory bowel disease and cancer. Additionally to the inflammatory diseases or those with an obvious inflammatory component, P2RX7 drugs showed efficacy in Azlheimer's and psychiatric disorders such as mania, depression and also prion disease (reviewed in Bartlett et al., 2014; Savio et al., 2018). Unfortunately, the precise localization of P2RX7 expression in the brain remains controversial due to non-specific antibody binding (Anderson and Nedergaard, 2006) with *p2rx7* KO mouse brains showing strong immunoreactivity (Sim et al., 2004). A recent progress in this area has been the development and initial characterization of a humanized P2RX7 mouse model, where hP2RX7 expression was localized to glutamatergic pyramidal neurons of the hippocampus, as well as to non-neuronal lineages including astrocytes, microglia, and oligodendrocytes (Metzger et al., 2017). Interestingly, in yet another model, the P2RX7_EGFP mice the receptor expression has been localized to the dentate gyrus granule cell layer (Jimenez-Pacheco et al., 2016). The existence of neuronal P2RX7 responses remains the topic of debate, with arguments for both the neuronal P2RX7 responses being an important pharmacological target (Miras-Portugal et al., 2017) but also suggesting that these responses are due to P2RX7 expression on astrocytes or microglia instead (Illes et al., 2017). Clearly, full understanding of the P2RX7 localisation in the brain remains elusive and would be required for the development of targeted therapeutic approaches for the variety of CNS diseases in which this receptor appears to play a role.

Despite these limitations, >70 patents for P2RX7 antagonists have been filed in the last few years, including for the use of A438079 in depression, anxiety and bipolar disorders, triazole-based drugs for CNS disorders and P2RX7 antagonists in epithelial cancers (from GSK, Abbott and Cleveland University Hospitals, respectively (Park and Kim, 2016). Moreover, trials have been conducted in relation to several inflammatory pathologies such as rheumatoid arthritis (Maini et al., 2006; Keystone et al., 2012), Crohn's (Eser et al., 2015), and basal cell carcinoma (Gilbert et al., 2017) to name but a few. Although AstraZeneca's AZD9056 trials were halted due to disappointing results, it's more recent application to Crohn's has been more promising. This suggests that P2RX7 inhibition may not be equally effective in various diseases with an inflammatory component and potentially that some drugs may show different efficacy in specific conditions. In this context, one remaining questions is whether selective targeting of the channel or the LP receptor functions would have more significant therapeutic effect. Another consideration is the impact of the P2RX7 polymorphisms, which are numerous and which have been demonstrated to impact various human disorders (Fuller et al., 2009). The benefit of the recent publication of the P2RX7 3-D crystal structure is that it will be possible to utilize it in the *in silico* high-throughput screening of chemical libraries and rational drug design. Structural prediction-based screening recently identified 3 novel competitive orthosteric antagonists. Intriguingly, one of these compounds was reported to specifically antagonize P2RX7 LP pore formation with no effect on the channel function (Caseley et al., 2016). Given that this drug binding to the eATP pocket affects LP only, it might provide some support for the hypothesis that the channel and pore are produced by the P2RX7 itself. However, while the publication focused on the therapeutic potential of this new “pore blocker” it did not consider this potentially distinguishing molecular mechanism.

Another example of the impact of the receptor crystal structure is the identification of the mechanism of action of AZT and other Nucleoside Reverse Transcriptase Inhibitors (NRTIs) at the P2RX7. These drugs have been shown previously to be able to block this receptor functions *in vitro* and to have therapeutic impact *in vivo*, in a number of mouse models of human diseases (Fowler et al., 2014). Molecular modeling showed that AZT and its derivatives occupy the same allosteric site as the canonical antagonists and also led to further work that proved AZT to be therapeutic in the mouse model of Duchenne muscular dystrophy (Al-Khalidi et al., 2018). AZT is a mainstay in the prevention of mother-to-child HIV transmission (Van Zyl et al., 2008). Consequently, there is extensive safety and pharmacokinetic data available following decades of AZT use in humans, also in children and neonates. Therefore, it is a good candidate for rapid re-purposing for treatment of human diseases requiring P2RX7 inhibition.

An exciting development in the P2RX7 targeting is a new class of antagonist—Multi-Target-Directed-Ligands (MTDLs), which permit the specific delivery of P2RX7 antagonists in a previously unattainably targeted manner. Specifically, dual NMDA-P2RX7 compounds were recently synthesized for targeting both the excitotoxicity-induced cell death as well as the P2RX7-dependent neuroinflammatory-induced cell death seen in Alzheimer's disease (Karoutzou et al., 2018). Such compounds represent an exciting and theoretically limitless combinatorial approach to P2RX7 inhibition.

With rational design-based drug screening set to replace classical approaches, the number of P2RX7 antagonists should increase further. Yet, for the rational clinical application of the existing as well as the new drugs, we still require a significant improvement of our basic understanding of underlying receptor physiology and pharmacology and of its involvement in various pathologies. Perhaps the holy grail of P2RX7 research remains the determination of the molecular mechanism of the channel vs. the LP opening (Karasawa et al., 2017) and also of the physiological and pathological importance of this phenomenon.

**Figure 3:**
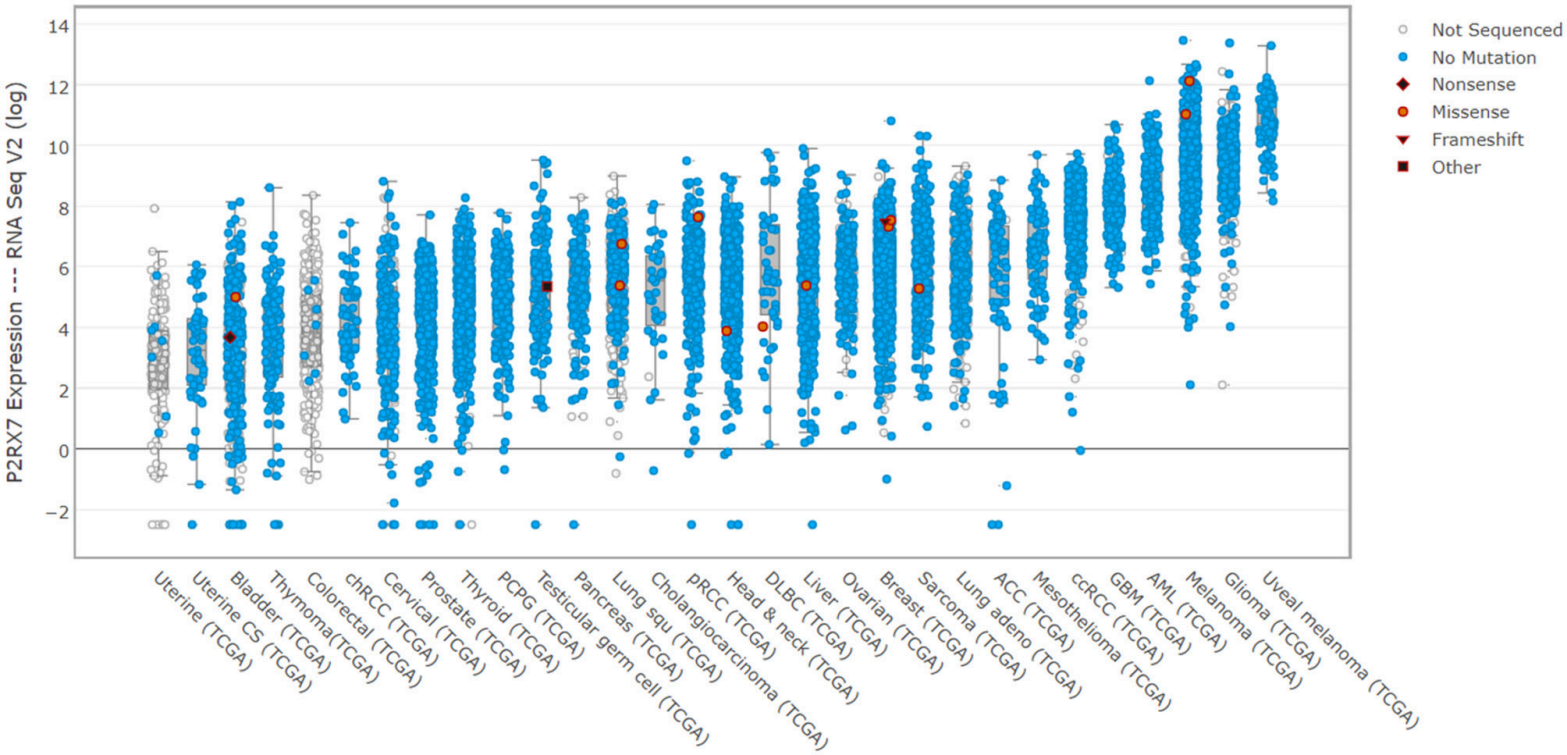
P2RX7 expression is elevated in human cancers. cBioPortal (cbioportal.org) derived expression levels for P2RX7 multiple human cancer patient biopsies from TCGA data (cancergenome.nih.gov). Noticeable, across almost all tumor types, is the significant overexpression of P2RX7 and a paucity of detected mutations. This indicates that malignant cells express high levels of fully-functional receptors, which is an important consideration for drug treatment.

Cancer is the paragon of P2RX7's dual nature. P2RX7 is ubiquitously overexpressed in cancer (Figure 3). Yet, tumor cells appear to take advantage of the receptor responses, without succumbing to the LP-mediated cell death cascade activation, which would be fully expected given such significant overexpression in the presence of abundant eATP in the tumor environment (Young et al., 2017). Quite the reverse, tumor cells use this P2RX7 overexpression to their significant advantage though increases in growth, migration, the Warburg effect, VEGF production triggering tumor neo-angiogenesis and MMP release helping its invasion and metastasis (Young et al., 2017; reviewed by Di Virgilio, 2016). While many tumors expressed the “non-functional” P2RX7, there is evidence that the functional receptor can be expressed too. Only few loss-of function mutations have been identified in P2X7 expressed in various tumor types (Figure 3). Thus, the systemic administration of pharmacological P2RX7 blockers should have a potent anti-tumor effect by preventing the high jacking of this receptor by cancer cells. Interestingly, while this approach showed promise in some tumors, the effect was far from universal. The problem is that P2RX7 is a significant player in the immune response that, in turn, can inhibit growth of some tumors (reviewed by Di Virgilio, 2016). Indeed, there are indications that immune suppression following P2RX7 inhibition is a real and potentially significant drawback, as tumors in *p2rx7* KO mice show accelerated growth, increased tumor size and metastasis, recruitment and invasion, all of which reflects reduced immune cell activation (Adinolfi et al., 2015). Perhaps the appropriate metaphor for P2RX7 in cancer would be that of the sword and the shield, where the sword may be blunted through receptor blockade but at the expense of lowering the shield hand. Hence, a successful cancer treatment with P2RX7 antagonists should, ideally, selectively target cancer cells whilst leaving the host immune response unscathed. In addition, a treatment exploiting the overexpression of P2RX7 on tumor cells and turning it against the tumor is an appealing proposition. The aforementioned targeting of the “non-functional” P2RX7 with specific antibodies is one example currently in clinical trials. Another could be the development of positive modulators of P2RX7 LP formation or compounds which effectively upregulate or reinstate P2RX7 LP formation in tumor cells, which overexpress either functional or “non-functional” receptor variants. Such an approach abrogates any concerns over immune response dampening and potentially could even boost the anti-tumor responses as certain types of cell death trigger or potentiate immune responses.

In conclusion, recent progress in obtaining the receptor crystal structure, understanding the mechanism of action of structurally unrelated antagonists and in unraveling the feedback mechanism regulating its functions should result in a breakthrough in targeting P2RX7 in a host of human diseases.

## Author contributions

CY and DG both made substantial, direct and intellectual contributions to the manuscript, and approved it for publication.

### Conflict of interest statement

Some aspects of research into P2RX7 antagonist as a treatment for Duchenne muscular dystrophy in DCG laboratory are funded by a research grant from Myotherix.

## Supplementary material

Video S1 P2RX7 chicken TNP-ATP RCSB PBD PyMOL movie.avi simulated 3D view of P2RX7 trimeric subunit configuration, showing ATP molecules bound.

## Abbreviations

eATP: extracellular Adenosine Triphosphate
BMDNs: Bone Marrow Derived Neutrophils
DAMPs: Danger Associated Molecular Patterns
PAMPs: Pathogen Associated Molecular Patterns
TLR: Toll-like receptor
NLRP3: NOD-like receptor protein 3
MMP: Matrix metalloproteinase
IL: Interleukin.
